# One-Chip Isolation of Drug-Resistant Acute Myeloid Leukemia Cells with CXCR4-Targeted Magnetic Fluorescent Nanoprobes

**DOI:** 10.3390/nano12101711

**Published:** 2022-05-17

**Authors:** Fan Wang, Yuqi Jiang, Luhai Wang, Yi Chen, Yu Zhang, Ming Ma

**Affiliations:** State Key Laboratory of Bioelectronics, Jiangsu Key Laboratory for Biomaterials and Devices, School of Biological Sciences and Medical Engineering, Southeast University, Nanjing 210096, China; 220191949@seu.edu.cn (F.W.); 213180878@seu.edu.cn (Y.J.); 230199077@seu.edu.cn (L.W.); 220174573@seu.edu.cn (Y.C.)

**Keywords:** acute myeloid leukemia, magnetic nanoparticles, nanoprobe, magnetic isolation, microfluidic chip

## Abstract

Drug resistance and relapse lead to high mortality in acute myeloid leukemia, and studies have shown that CXCR4 overexpression is highly correlated with poor prognosis and drug resistance in leukemia cells. Isolation and detection of AML cells with CXCR4 overexpression will be crucial to the treatment of AML. In this paper, magnetic nanoparticles were firstly prepared successfully by high-temperature thermal decomposition method, and then characterized by TEM, VSM and DLS. Subsequently CXCR4-targeted magnetic fluorescent nanoprobes conjugated with antibody 12G5 were constructed by stepwise coupling. In cell experiments, the obtained probes demonstrated excellent targeting efficacy to CXCR4 overexpressed AML cells HL-60. In addition, HL-60 cells labelled with the magnetic probes can be magnetic isolated successfully in one microfluidics chip, with efficiency of 82.92 ± 7.03%. Overall, this method utilizes the superiority of superparamagnetic nanomaterials and microfluidic technology to achieve the enrichment and capture of drug-resistant cells in a microfluidic chip, providing a new idea for the isolation and detective of drug-resistant acute myeloid leukemia cells.

## 1. Introduction

Acute myelogenous leukemia (AML) is a common malignancy of the hematopoietic system with a high mortality rate [1,2]. This phenomenon is mainly due to the fact that the treatment of AML is still dominated by chemotherapy with broad-spectrum drugs, which can easily lead to drug resistance and relapse [3,4,5]. Previous studies have shown that the interaction between the bone marrow microenvironment and AML cells is important for chemoresistance and disease relapse, and that the CXC chemokine receptor 4 (CXCR4) and CXC chemokine ligand 12 play important roles in regulating the interaction between the bone marrow microenvironment and leukemic cells [6,7,8,9,10]. Furthermore, several researches evaluated the possible correlation between prognosis and CXCR4 expression in AML patients. Ploemacher et al. investigated CXCR4 expression in adult AML patients and found that patients with high CXCR4 expression had a significantly lower survival rate and a higher likelihood of relapse, with a median relapse-free survival of 8.3 months [11]. Guyotat et al. evaluated the prognostic significance of CXCR4 in 90 AML patients with flow cytometry and showed that patients with low CXCR4 expression had a better prognosis and longer relapse-free time, with an overall survival rate of 24.3 ± 2.9 months compared to 12.8 ± 2 months for patients with high CXCR4 expression [12]. CXCR4 overexpression has been shown to be a poor prognostic factor in AML and patients with high CXCR4 expression have a poorer prognosis and a higher likelihood of drug resistance and relapse. Meanwhile, CXCR4 is an important target for drug resistance in AML, and CXCR4 overexpression can be used as a biosignal to express resistance, relapse, and poor prognosis in AML [13,14,15]. Therefore, CXCR4 overexpression could be an important biological signal for drug resistance in AML. The key technology to address AML drug resistance is to achieve the isolation and detection of drug-resistant cells [16,17].

Currently, magnetic nanoparticles (MNPs) have unique properties in magnetic targeting, magnetic resonance imaging, chemotherapy, thermotherapy, bioseparation, gene therapy, enzyme immobilization, and drug release, etc. [18]. Wang et al. detected fecal k-ras mutations in different stages of pancreatic cancer by constructing magnetic fluorescent nanoprobes [19]. Karimi et al. used magnetic nanoprobes to separate specific stem cells and leukocytes from cell suspensions [20]. Magnetic iron oxide nanoparticles have attracted much attention due to their low toxicity, biodegradability and excellent magnetic properties, etc. [21]. At present, the mainstream cell sorting methods are flow cytometry and magnetically activated cell sorting, but flow cytometry requires more expensive instruments, and magnetically activated cell sorting requires large loading volume and low separation efficiency, both of which have their disadvantages [22,23]. With the promotion of microfluidics, the strength of small sample volume, reagent savings, low cost, and high integration coincide with the need for cell sorting, and there are more and more methods for sorting cells based on microfluidics [24]. Myklatun et al. found that macrophages containing engulfed magnetic nanoparticles could be sorted with an efficiency of 90 ± 1% [25]. Ozkumar et al. successfully used microfluidic technique to separate magnetically labeled CTCs and leukocytes from blood [26]. In brief, several studies show the great potential of the combination of magnetic separation technology and microfluidics.

**Scheme 1:**
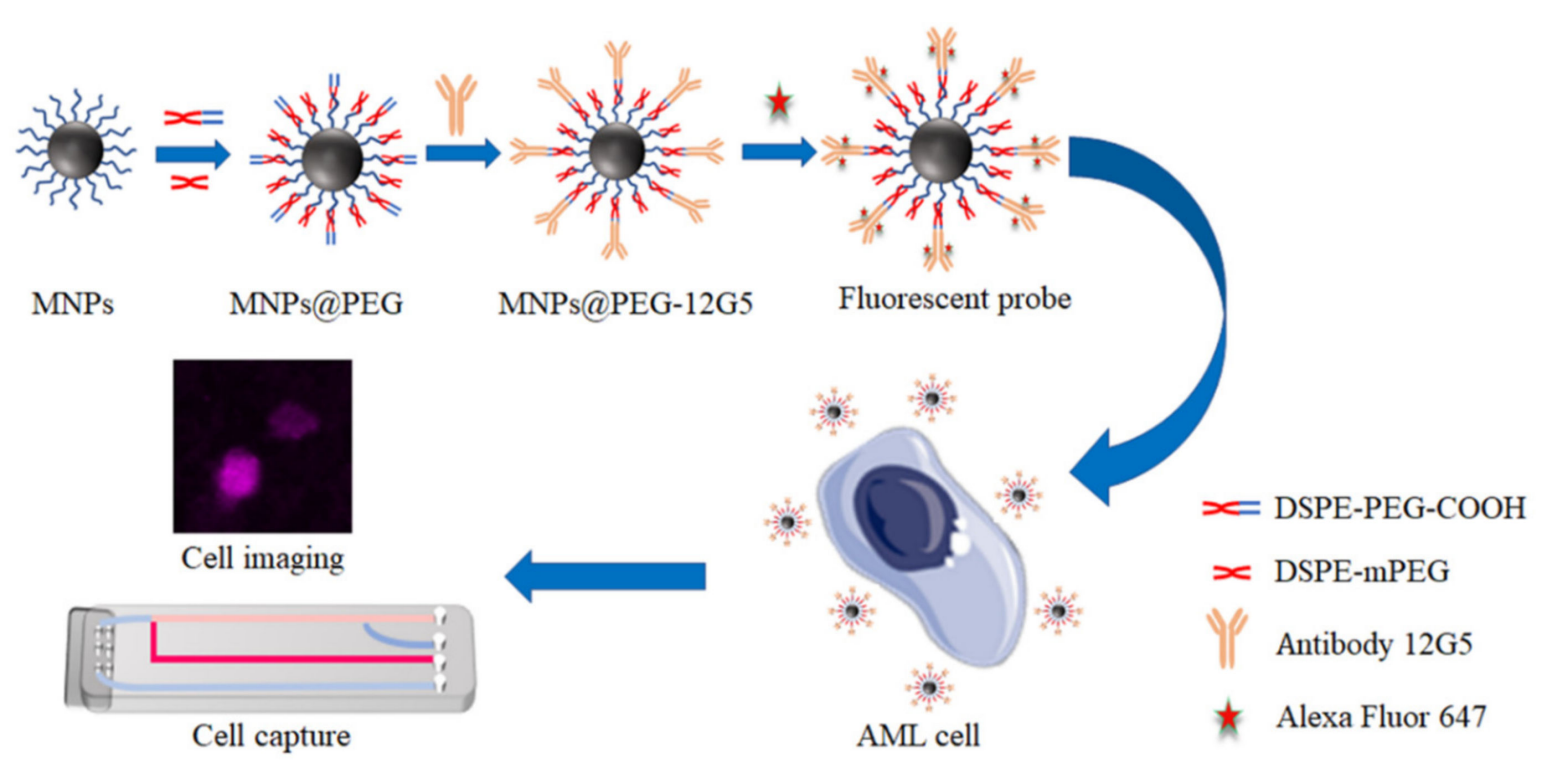
Schematic illustration of the procedure for fabrication and application of magnetic fluorescent nanoprobes.

Nowadays, many CXCR4 probes have been constructed for imaging or therapeutic purposes, and fewer reports have used this probe to achieve the isolation and detection of drug-resistant cells [27,28]. Flow cytometric sorting is a very effective means, but flow cytometry, as a bulky precision instrument, cannot meet the demand for portable and rapid cell separation assays [29]. Magnetic sorting using magnetic beads is another traditional method for isolating cell populations from biological suspensions, but usually requires higher sample sizes [30]. Herein, we constructed a magnetic fluorescent nanoprobe that can target CXCR4. As shown in Scheme 1, the carboxylic polyethylene glycol (PEG) is used to stabilize the MNP dispersion in the aqueous phase and to prevent the non-specific binding [31]. Antibodies 12G5 are conjugated on the MNP@12G5 surface for specific targeting CXCR4 pathway [32], subsequently fluorescent dye Alexa Fluor 647 is conjugated to 12G5 for cell imaging. The magnetic probes will be used to specific label AML cell line HL-60, and achieve magnetic cell isolation on a microfluidics chip.

## 2. Materials and Methods

### 2.1. Experimental Materials

Iron acetylacetonate (Fe(acac)_3_, purity > 97%) was purchased from Sigma Aldrich, Taufkirchen, Germany. Oleic acid (purity > 85%) was purchased from Shanghai Aladdin Reagent Company, Shanghai, China. Dibenzyl ether (purity > 98%) was purchased from Alfa Aesar Chemicals. DSPE-mPEG2000 (purity > 99%) was obtained from Shanghai Aviator Pharmaceutical Technology Co., Shanghai, China. DSPE-PEG2000-COOH (purity > 99%) was obtained from Xi’an Ruixi Biotechnology Co., Xi’an, China. CXCR4 antibody (12G5) was purchased from R&D systems. Alexa Fluor 647 was obtained from Thermo Fisher Scientific, Shanghai, China.

### 2.2. The Synthesis and Characteration of MNP@PEG

Iron oxide nanoparticles of suitable size were prepared by using high-temperature thermal decomposition method and the samples were preserved in trichloromethane solvent [33]. In detail, 20 mmoL Fe(acac)_3,_ 100 mL dibenzyl ether and 23 mL oleic acid were mixed and put into a three-neck round-bottom flask. Slowly passing a certain rate of nitrogen gas, programmed heating was performed under condensing reflux conditions for 3 h. After the reaction, the morphology and size of MNPs were characterized by transmission electron microscopy (TEM, JEOL, Akishima, Japan). Vibrating sample magnetometer (VSM, Lakeshore 7407, Columbus, OH, USA) was used to characterize the sample magnetism at room temperature, and its saturation magnetization intensity was calculated.

PEG was modified on the surface of MNP by rotary evaporation method to obtain stable MNP@PEG in aqueous solution. Both 150 mg DSPE-mPEG2000 and 50 mg DSPE-PEG2000-COOH were dissolved in 4 mL of trichloromethane and put into a 50 mL single-necked round-bottom flask. Then trichloromethane solution containing 10 mg of MNP was added and 4 mL of diluted water was added. The mixed solution was rotary evaporated in a water bath at 70 °C for about 20 min to fully remove the trichloromethane. After modification of PEG, MNP@PEG was purified by magnetic separation column (Nanoeast Biotech, Nanjing, China). Dynamic light scattering (DLS, Zetasizer ZS90, Malvern, UK) was used to measure hydrodynamic size and zeta potential of MNP@PEG (3 measurements per group).

### 2.3. The Synthesis of MNP@PEG-12G5-F647

1 mg of MNP@PEG solution and 2 mL of MES (0.01 M, pH 5.5) were mixed with 100 µL and 200 µL of 12G5 antibody (dissolved in PBS with the concentration of 0.5 mg/mL), respectively. The mixed solution was shaken on an oscillator for 30 min (25 °C, 120 rpm). Then 500 µL EDC (dissolved in PBS with concentration of 1 mg/mL) was added into the solution and shaken for 4.5 h at 25 °C. After reaction, MNP@PEG-12G5 was purified by magnetic separation column.

To conjugate F647 to MNP@PEG-12G5, a certain proportion of F647 (dissolved in DMSO with the concentration of 10 mg/mL), MNP@PEG-12G5 and 2 mL of BBS buffer (0.02 M, pH 8.0) were mixed up for 1 h on an oscillator (25 °C, 120 rpm). After that, magnetic separation column was used for purification to obtain MNP@PEG-12G5-F647. DLS was used to measure hydrodynamic size and zeta potential of the sample (3 measurements per group) and fluorescence spectrophotometer (FluoroMax-4, Horiba Co., Kyoto, Japan) was used to measure fluorescence intensity of the probes.

### 2.4. Cell Culture

Cell line HL-60 (human acute promyelocytic leukemia cell) used in the experiment was obtained from Jiangsu KGI Bio and grown in modified IMDM medium containing 20% fetal bovine serum. MS-5 cells were grown in incomplete DMEM (high glucose) medium containing 10% fetal bovine serum (double antibody). The cells were all grown in the incubator containing 5% CO_2_ at 37 °C.

### 2.5. Cell Labeling with 12G5-F647 and MNP@PEG-12G5-F647

Fluorescent antibodies 12G5-F647 was first prepared by conjugating antibody 12G5 with fluorescent molecules F647. 12G5-F647 was then added into 250 µL of PBS (0.01 M, pH 7.4) containing 5 × 10^5^ HL-60 cells or MS-5 cells, respectively. After incubation for 24 h at room temperature, an appropriate volume of the above samples was taken out for cell shaking, by which most of the cells were adhered to the slides. Then DAPI staining solution was added dropwise and stained for 5 min. The running water was used to rinse the slides and wipe off with absorbent paper. The cells were observed under the confocal microscopy and the remaining samples were quantified by using flow cytometry to discern the expression level of CXCR4 on the cell surface.

To evaluate the targeting effect of the previously synthesized MNP@PEG@12G5-F647, 20 µg nanoprobes were added to 5 × 10^5^ fixed HL-60 cells and incubated overnight at 37 °C on an oscillator. Similarly, samples were taken out for cell shaking. Then the DAPI staining solution was added and stained for 5 min. Confocal microscopy and flow cytometry were used to observe separately.

By considering the effects of different probe amounts, different total volume amounts and incubation times in order to clarify the conditions of use of MNP@PEG@12G5-F647. Different amounts of MNP@PEG@12G5-F647 were added into 250 µL of PBS containing 5 × 10^5^ HL-60 cells and incubated for 0.5 h at 37 °C for quantitative detection using flow cytometry. Based on the above results, the appropriate number of probes were added into different volumes of PBS containing 5 × 10^5^ HL-60 cells and incubated for 0.5 h at 37 °C for quantitative detection using flow cytometry. Similarly, different incubation times were adjusted according to the above conditions and the results were measured by flow cytometry.

### 2.6. Microfluidic Array Fabrication

This experiment adopted Auto CAD 2020 genuine software to realize the design of microfluidic chip, and contacted a professional company (Suzhou Boaz Microfluidics Co., Ltd., Suzhou, China) to help processing. The chip was in the form of PDMS bonded glass, the size of PDMS was 90 × 10 × 3 mm, the thickness of glass was 1 mm, and the uniform depth of flow channel was 40 µm.

### 2.7. Cell Capture

Magnetic separation column and microfluidics chip were used to capture the cells labelled by MNP@PEG-12G5-F647, respectively. The labeled cell PBS solution was added to the magnetic separation column for magnetic separation, and the cell concentration in the filtrate was measured by counting with a cell counter (CountessⅡ, Thermo Fisher Scientific Co., Shanghai, China), and the cell separation efficiency was calculated according to the formula:

Cell separation efficiency (%) = (1-Fc × Fv/N) × 100;

Fc = Concentration of cells in the filtrate;

Fv = Volume in the filtrate;

N = Total number of cells added.

**Figure 1:**
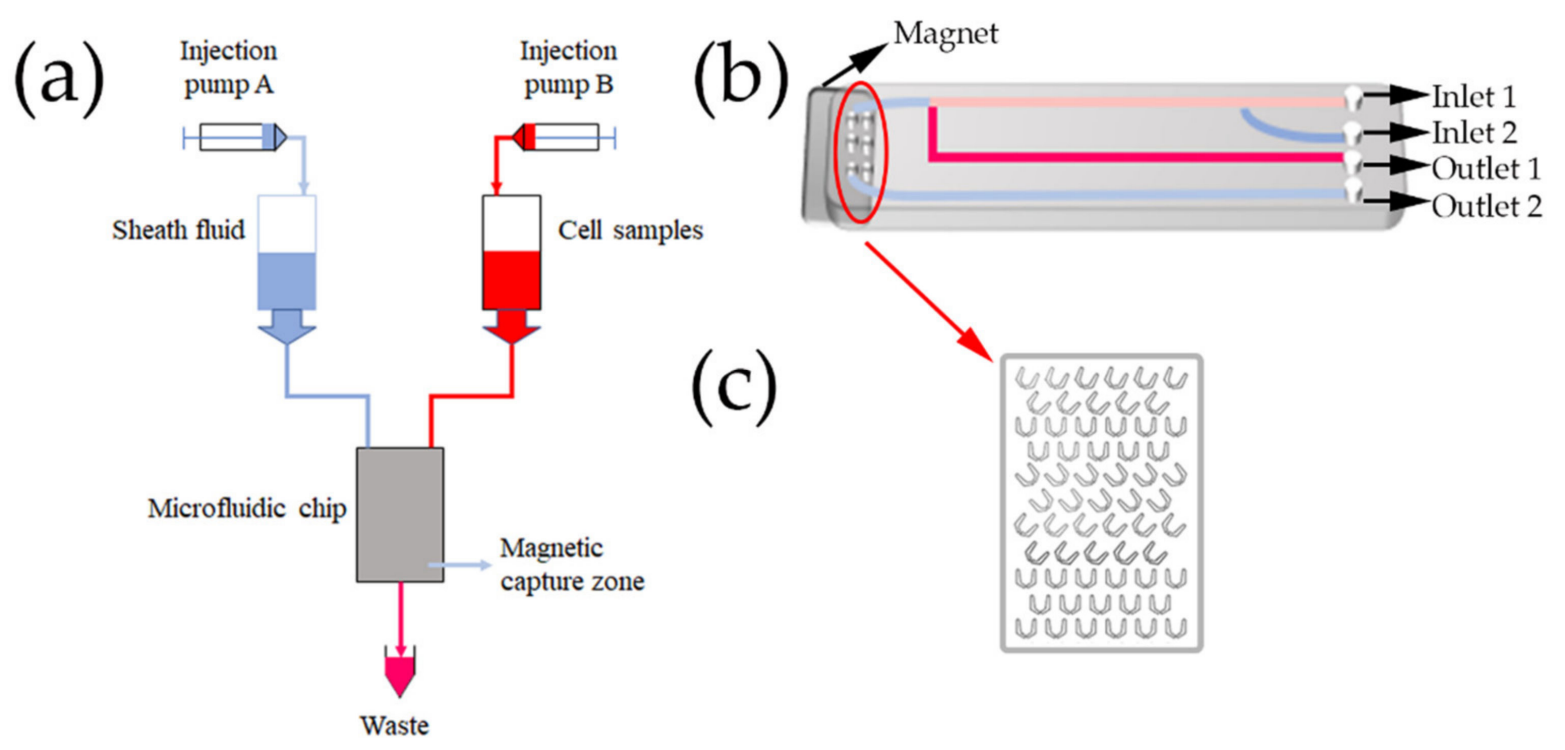
Schematic of magnetic capture microfluidic setup (**a**), microfluidic chip, (**b**) and magnetic capture zone (**c**) in the chip.

The schematic of cell capture in microfluidics chip is shown in Figure 1a. Two syringe pumps were used to control the flow rate of the sheath fluid and cell samples, respectively [34]. The fluid was pumped into the microfluidic chip by the syringe pumps and the waste fluid at the outlet was first collected without applying a magnetic field. The magnetic field was then applied to the U-shaped array region in the microfluidic chip to collect the waste fluid at the outlet, as shown in Figure 1b. The target cells were captured in the U-shaped array region of Figure 1c. The cell counter was used to calculate the cells collected at the outlet and calculate the cell separation efficiency on the microfluidic chip. The experiment was repeated three times and the magnetic capture rate and error bars were calculated.

## 3. Results and Discussion

### 3.1. Characterization of Magnetic Fluorescent Nanoprobes

**Figure 2:**
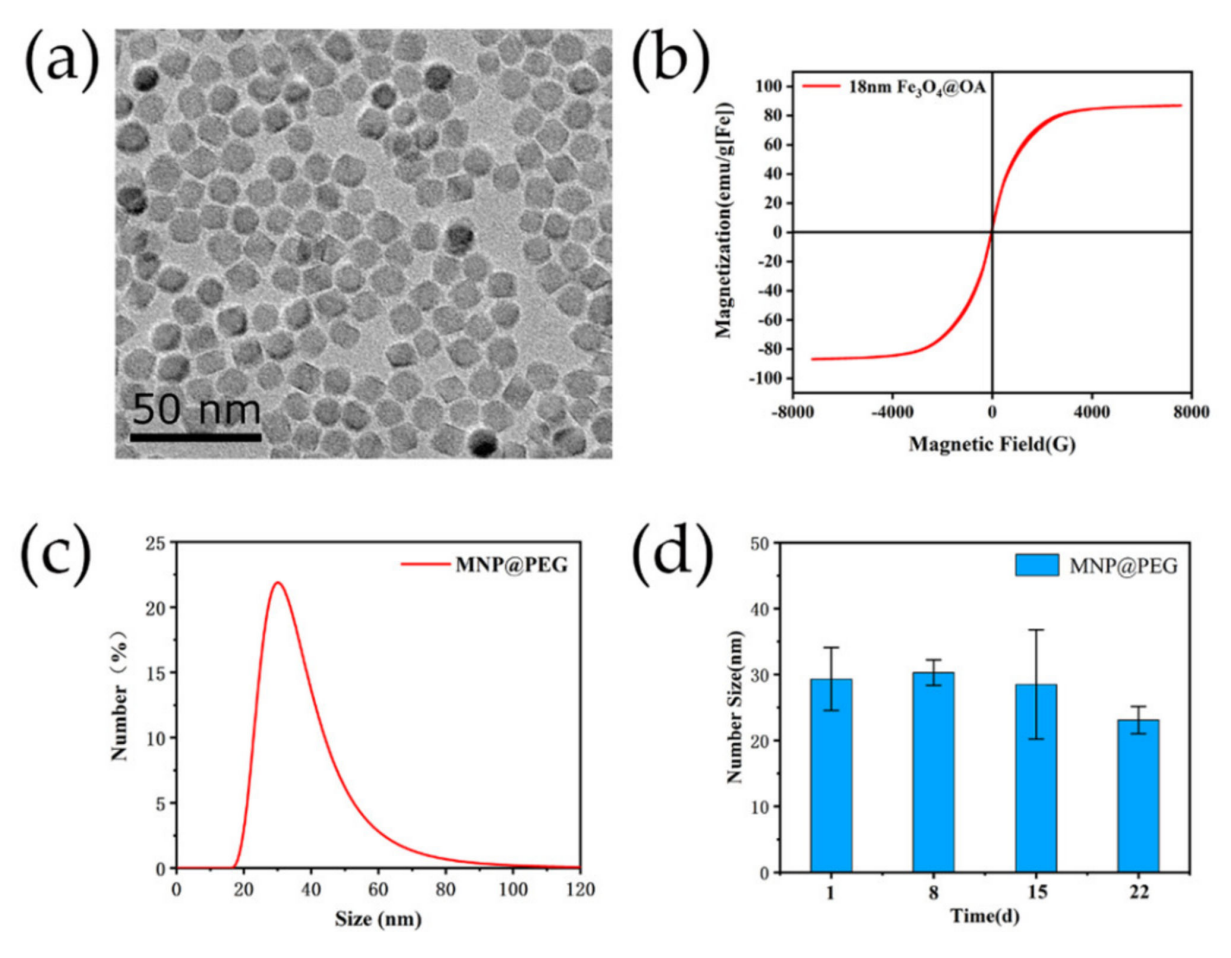
(**a**) A TEM image of iron nanoparticles. (**b**) Field-dependent magnetic hysteresis loops of iron nanoparticles at room temperature. (**c**) Hydrodynamic dimensions of MNP@PEG. (**d**) Stability of MNP@PEG.

By adjusting the flow rate of nitrogen and the amount of oleic acid, MNPs were successfully synthesized through high-temperature thermal decomposition method. Based on the TEM characterization (Figure 2a), the MNPs with high yield show regular morphology, excellent dispersion and uniform size distribution. Additionally, the diameter of MNPs (18.17 ± 1.69 nm) was measured by using the Image J software. To investigate the magnetic properties of the samples, the solid Fe_3_O_4_@OA nanoparticles were further magnetically characterized with VSM. As the results shown in Figure 2b, both the remanence and coercivity of the prepared nanoparticles are approximately equal to zero, demonstrating their excellent superparamagnetic properties. In addition, their saturation magnetization was calculated with a high value of 86.81 emu/g[Fe]. Therefore, the MNPs with better magnetic properties can be used as a suitable candidate for the following magnetic capture of cells.

However, the MNPs dissolved in organic solutions were not suitable for biomedical applications. The surface of MNPs can be surrounded by DSPE-mPEG with high biocompatibility through hydrophobic interaction, forming a stable phospholipid monomolecular layer with carboxyl-terminal sites on the surface of MNP@PEG. Such phospholipid monomolecular can not only contribute to stable dispersion in the trans-aqueous phase, but also help to prevent non-specific binding. As shown in Figure 2c, the hydrodynamic size of the MNP@PEG is 29.33 ± 4.75 nm, and the zeta potential is −42.2 ± 1.16 mV, which indicates an ideal dispersion of MNP@PEG. Moreover, the hydrodynamic size of the MNP@PEG basically remained the same even after three weeks (Figure 2d), revealing its admirable stability in aqueous phase.

Since it was necessary to optimize the antibody feeding amount, fluorescent magnetic nanoprobes with different antibody feeding amounts were prepared. For sample MNP@PEG-12G5-L-F647, it had an antibody dose of 50 µg, and for sample MNP@PEG-12G5-H-F647, the antibody dose was 100 µg. The amount of antibody in the filtrate was quantified using the BCA kit, and then the quantity of the antibody coupling was calculated by subtracting both from the amount of antibody added. Similarly, the number of fluorescent molecules in the filtrate was calculated by measuring the absorbance of the filtrate at 649 nm, and thus the number of fluorescent molecules coupled was obtained. The coupling quantity and coupling ratio of the antibody and fluorescent molecules were measured and shown in Table 1. The antibody coupling rate was the same for both groups, but the antibody coupling amount was higher for sample MNP@PEG-12G5-H-F647.

**Figure 3:**
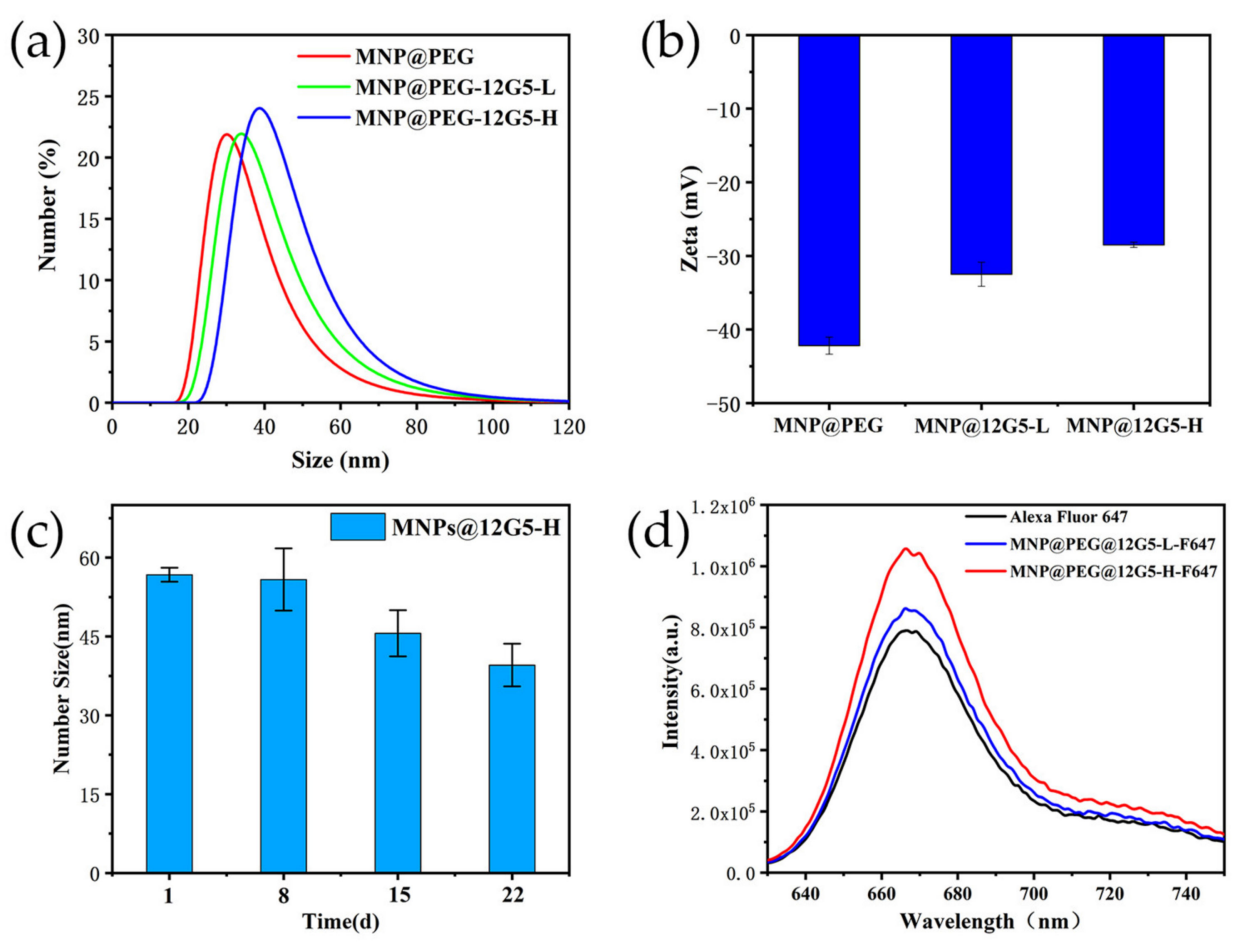
Hydrodynamic distribution (**a**), zeta potential (**b**), and storage stability (**c**) of MNP@PEG-12G5. (**d**) The fluorescence emission spectrum of MNP@PEG-12G5-F647. (The excitation wavelength was 620 nm and the emission wavelength was 630 nm~700 nm.).

Furthermore, hydrodynamic size, surface zeta potential, stability and fluorescence intensity of the two samples with different amounts of antibody and fluorescent molecule coupling were investigated. As shown in Figure 3a, hydrodynamic size of MNP@PEG-12G5-H-F647 was slightly larger than that of MNP@PEG-12G5-L-F647 since more antibodies were coupled, but the change in hydrodynamic size was not very obvious. However, the change in surface zeta potential was very obvious, as shown in Figure 3b, the surface zeta potential of MNP was −42 mV, −32 mV for MNP@PEG-12G5-L-F647 and −28 mV for MNP@PEG-12G5-H-F647. The reason for the change in potential is mainly that the carboxyl site of PEG was occupied, and the success of antibody coupling can be clearly demonstrated by the change in potential. After the dispersion was placed for a period of time, a part of the particles with large hydrodynamic size aggregated or precipitated, and the particles with smaller size were still dispersed in the supernatant, which could explain the decrease of the average hydrodynamic size (Figure 3c).

With coupling of the fluorescent molecule F647, the fluorescence emission spectra of the samples were measured using a fluorescence spectrophotometer. As shown in Figure 3d, it can be concluded from the waveforms in the figure that both groups were successfully coupled the F647 fluorescent molecule so that a similar waveform could be observed. In addition, since the samples were detected by fluorescence emission spectrum contained the same amount of iron, the magnitude of their fluorescence intensity can be evaluated according to the waveform peaks, and it is obvious that the fluorescence intensity of sample MNP@PEG-12G5-H-F647 was stronger. It was due to the fact that more antibodies were coupled to this group, resulting in a high quantity of fluorescent molecules coupled, and the fluorescence intensity of sample MNP@PEG-12G5-H-F647 was stronger at the same iron concentration, so this result was also in accordance with our expectation.

### 3.2. Cell Labeling with 12G5-F647 and MNP@PEG -12G5-F647

**Figure 4:**
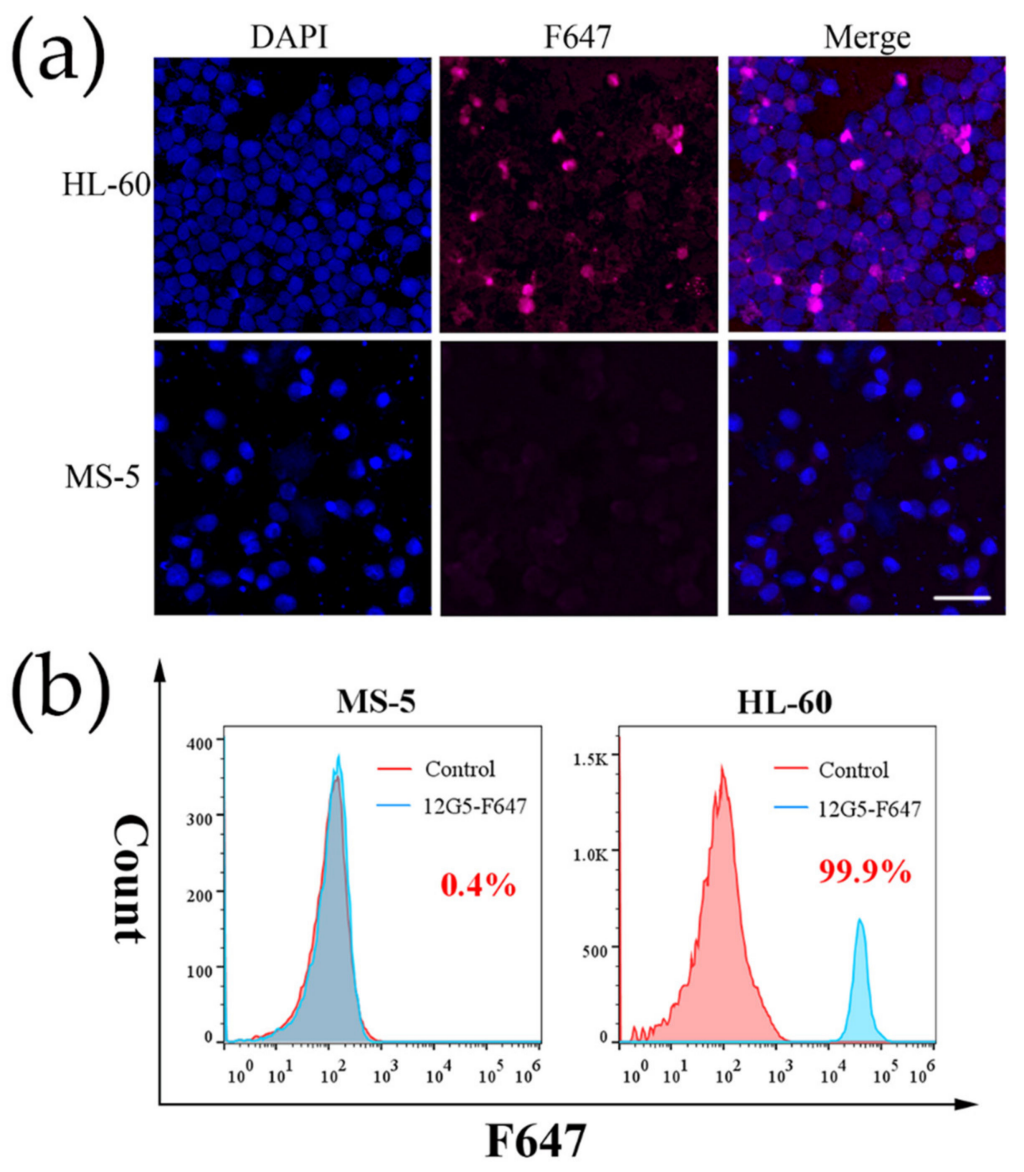
CXCR4 expression levels in cells. (**a**) The laser confocal images of HL-60 and MS-5 cells incubated with fluorescent antibody, the left, middle and right show DAPI (blue), F647 (pink) and the merged image, respectively. Scale bar: 30 µm. (**b**) Flow cytometry detection of CXCR4 expression levels in MS-5 and HL-60 cells after incubation for 24 h at room temperature.

12G5 is the most commonly studied monoclonal antibody that binds specifically to CXCR4 and can be used to detect the expression level of CXCR4 on the cell surface. To evaluate the expression of CXCR4 antigen on the cell surface, cells were co-incubated with antibody 12G5 conjugated with fluorescent molecules and the level of CXCR4 receptor expression on the surface of HL-60 and MS-5 cells was measured using confocal microscopy and flow cytometry. As illustrated in Figure 4a, there was fluorescence of F647 on HL-60 cells with pink color, while there was almost no such fluorescence on MS-5 cells, which visually demonstrated that HL-60 cells expressed CXCR4 antigen, while MS-5 cells hardly expressed it. To confirm this finding, flow cytometry was performed on both cells, and the results are shown in Figure 4b, further demonstrating the view that MS-5 cells barely express CXCR4 antigen while HL-60 cells highly express CXCR4 antigen [35].

**Figure 5:**
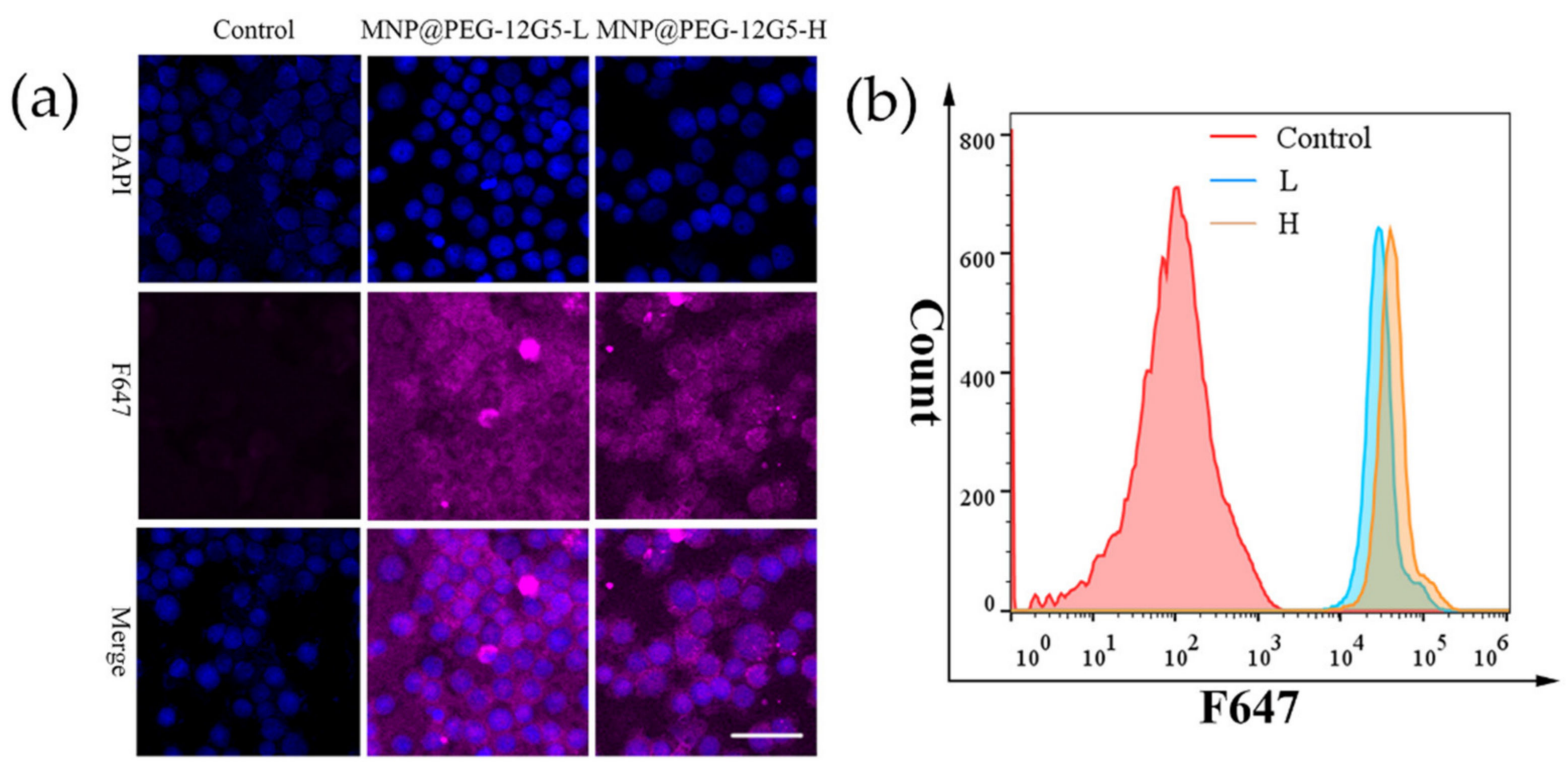
(**a**) The laser confocal images of HL-60 cells incubated with MNP@PEG-12G5-L and MNP@PEG-12G5-H, the up, middle and down show DAPI (blue), F647 (pink) and the merged image, respectively. Scale bar: 30 µm. (**b**) Flow cytometry detection of CXCR4 expression levels in HL-60 cells after incubation with MNP@PEG-12G5-L and MNP@PEG-12G5-H for 0.5 h at 37 °C.

To evaluate the targeting specificity of the magnetic fluorescent nanoprobe, HL-60 cells were first co-incubated with the probe and observed under confocal microscopy after the same incubation time. As shown in Figure 5a, both the groups H and L, the cells could be clearly seen to present a ring of pink fluorescence, which means that the probe was able to target the cells accurately. However, the background interference was significant in group L. Therefore, the results of the flow cytometry were further compared between the two groups, and the results are shown in Figure 5b. Either the probe MNP@PEG-12G5-H-F647 or MNP@PEG-12G5-L-F647 could successfully target the HL-60 cells with CXCR4 overexpression. The quantity of fluorescent molecules coupled to MNP@PEG-12G5-H-F647 was more, and the fluorescence intensity was higher, resulting in stronger fluorescence intensity in the H group of cells. Due to the limited number of binding sites on the surface of HL-60 cells, the average fluorescence intensity of the cells using the probes in group H was significantly stronger after sufficient conjugation. Since the eventual purpose of this work is to isolate and capture cells, it is desirable that the fluorescence intensity of individual cells be stronger, which will help improve the capture efficiency. In the subsequent sections, if not specifically stated, all probe synthesis ratios for group H would be taken and experiments would be performed using that probe.

The ability of the probe to target cells is closely related to conditions such as probe concentration and incubation time [36]. For clarifying the conditions of use of the obtained magnetic fluorescent nanoprobes, we successively explored the effects of the amount of probe used, the total volume of incubation and the incubation time on the targeting efficiency of the probes.

**Figure 6:**
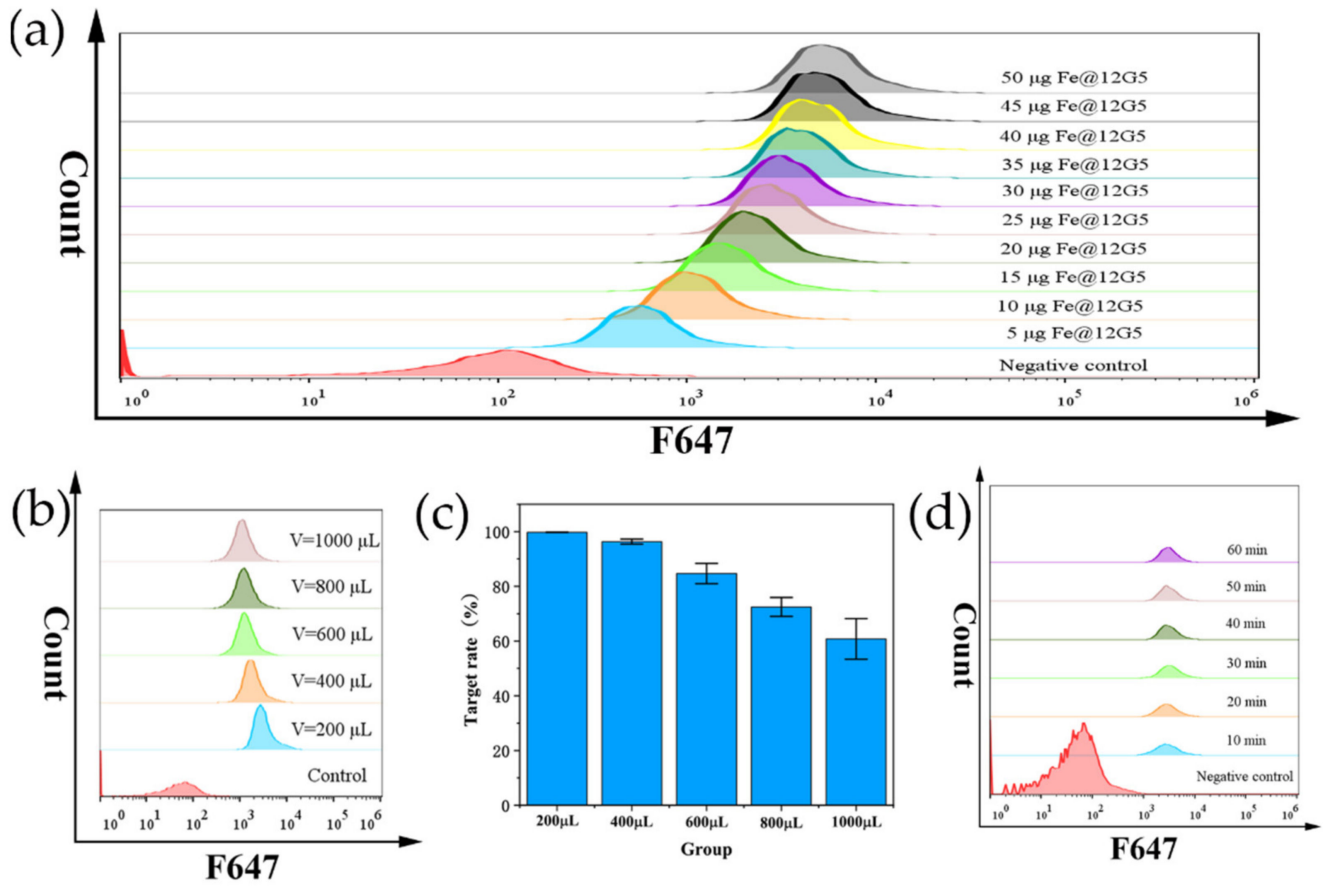
The quantitative analysis of magnetic fluorescent nanoprobe targeting efficiency by flow cytometry. (**a**) 5, 10, 15, 20, 25, 30, 35, 40, 45, and 50 µg of magnetic fluorescent nanoprobes were added into 250 µL PBS solution containing 5 × 10^5^ HL-60 cells and incubated for 0.5 h at 37 °C, respectively. (**b**) 30 µg of magnetic fluorescent nanoprobes were added to 200, 400, 600, 800, and 1000 µL of PBS solution containing 5 × 10^5^ HL-60 cells, respectively, and incubated for 0.5 h at 37 °C. (**c**) Statistics on the targeting efficiency of probes at different volumes. (**d**) 30 µg of magnetic fluorescent nanoprobes were added to 250 µL of PBS solution containing 5 × 10^5^ HL-60 cells and incubated for 10, 20, 30, 40, 50, 60 min, respectively, at 37 °C.

First, magnetic fluorescent nanoprobes with a mass range from 5 to 50 µg (in terms of Fe element) were added to the PBS solution containing 5 × 10^5^ HL-60 cells. As shown in Figure 6a, the average fluorescence intensity of the cells slowly increased with the amount of magnetic fluorescent nanoprobes, but the change began to gradually slow down after the addition of 30 µg, at which point close to 100% capture rate was achieved. Therefore, 30 µg of magnetic fluorescent nanoprobe was the optimal dosage. Although the capture rate was affected by the amount of probe affects the capture rate, it is intrinsically related to the concentration on the capture efficiency. When a certain amount of probe was added, the concentration depends inversely on the total volume. From the microfluidic point of view, the volume was actually related to the injection speed, since the sample injection should be completed within the specified time. With a larger sample volume, the rate of injection needs to be accelerated, and too fast an injection rate would be prone to leakage from the microfluidic chip. So, the probe usage of 30 µg was selected and added to 200, 400, 600, 800, and 1000 µL of PBS solution containing 5 × 10^5^ HL-60 cells, and the detailed results are shown in Figure 6b,c. As can be seen with the increase of volume, the probe targeting effect was gradually decreasing as the volume increased, and when the volume was in the range of 200~400 µL, its targeting efficiency varied slightly. Considering that there might be residual liquid in the catheter when the microfluidic chip was used, the optimal total volume of incubation was set to 250 µL. The incubation time not only had an impact on the targeting efficiency of the probe, but also on the overall cell capture speed. Probes were added to 5 × 10^5^ fixed HL-60 cells and supplemented with PBS solution to 250 µL and incubated for 10, 20, 30, 40, 50, and 60 min at 37 °C. The results of flow cytometry detection are shown in Figure 6d. It can be clearly demonstrated that the targeting efficiency of the probe had reached more than ninety percent after 10 min of incubation with HL-60 cells. With the increase of time, the overall average fluorescence intensity of the cells enhanced, but the enhancement effect would not be particularly obvious even after 60 min of incubation. Therefore, the incubation time of 20 min was chosen, and the targeting rate in this state was almost 100%, and the average fluorescence intensity of the cells was also at a high level.

### 3.3. Cell Capture

**Figure 7:**
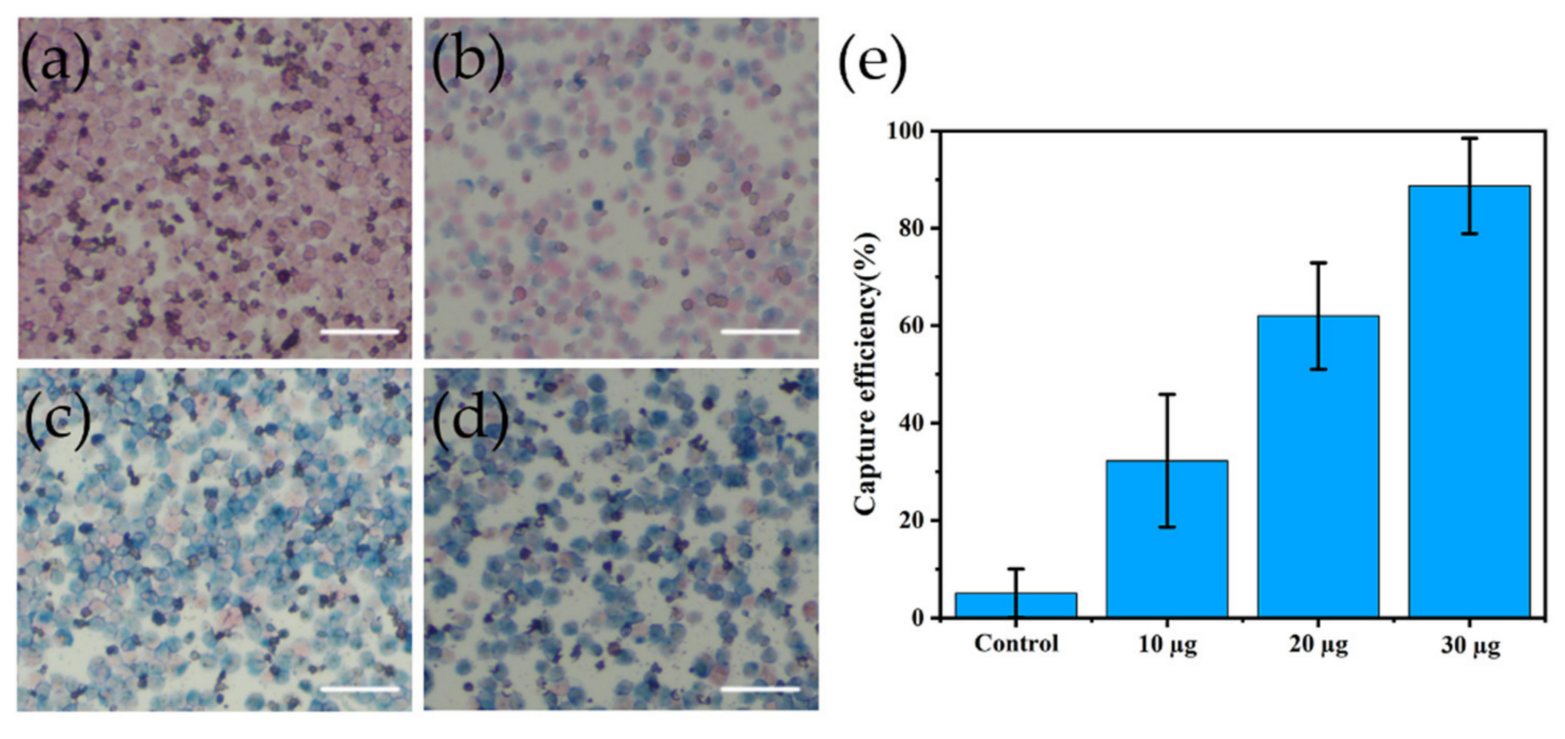
Targeting and magnetic capture ability of nanoprobes. (**a**–**d**) Targeting ability testing of 0, 10, 20, 30 µg nanoprobes, respectively, by Prussian blue staining on HL-60 cells. Scale bar: 50 µm. (**e**) Cell magnetic capture efficiency calculated separately by adding 0, 10, 20, and 30 µg nanoprobes, respectively.

After targeting the magnetic fluorescent nanoprobe to the cells, the magnetic capture efficiency was explored by utilizing its superparamagnetic characteristics to achieve the targeted cell capturing effect [37]. According to previous experiments, the probe was able to accurately capture HL-60 cells. The target cells can be captured when a magnetic field as applied. 10, 20, and 30 µg of MNP@PEG-12G5-F647 were added to PBS solution containing HL-60 cells and incubated at 37 °C for 20 min. The magnetic fluorescent nanoprobe released Fe^3+^ upon interaction with hydrochloric acid, which will combine with ferricyanide ions to produce Prussian blue precipitation, appearing blue, as shown in Figure 7a–d. With the increase of the probe usage, the more cells bound to the probe, the blue staining of the cells gradually deepened. Such samples were subjected to magnetic separation operation, passed through the magnetic separation column separately, and the magnetic capture efficiency was calculated by measuring the number of cells in the filtrate. The results are shown in Figure 7e and the magnetic capture efficiency was also at a high level under the optimal conditions of the probe, and the magnetic capture efficiency of 88.71 ± 9.80% could be reached through the magnetic separation column.

**Figure 8:**
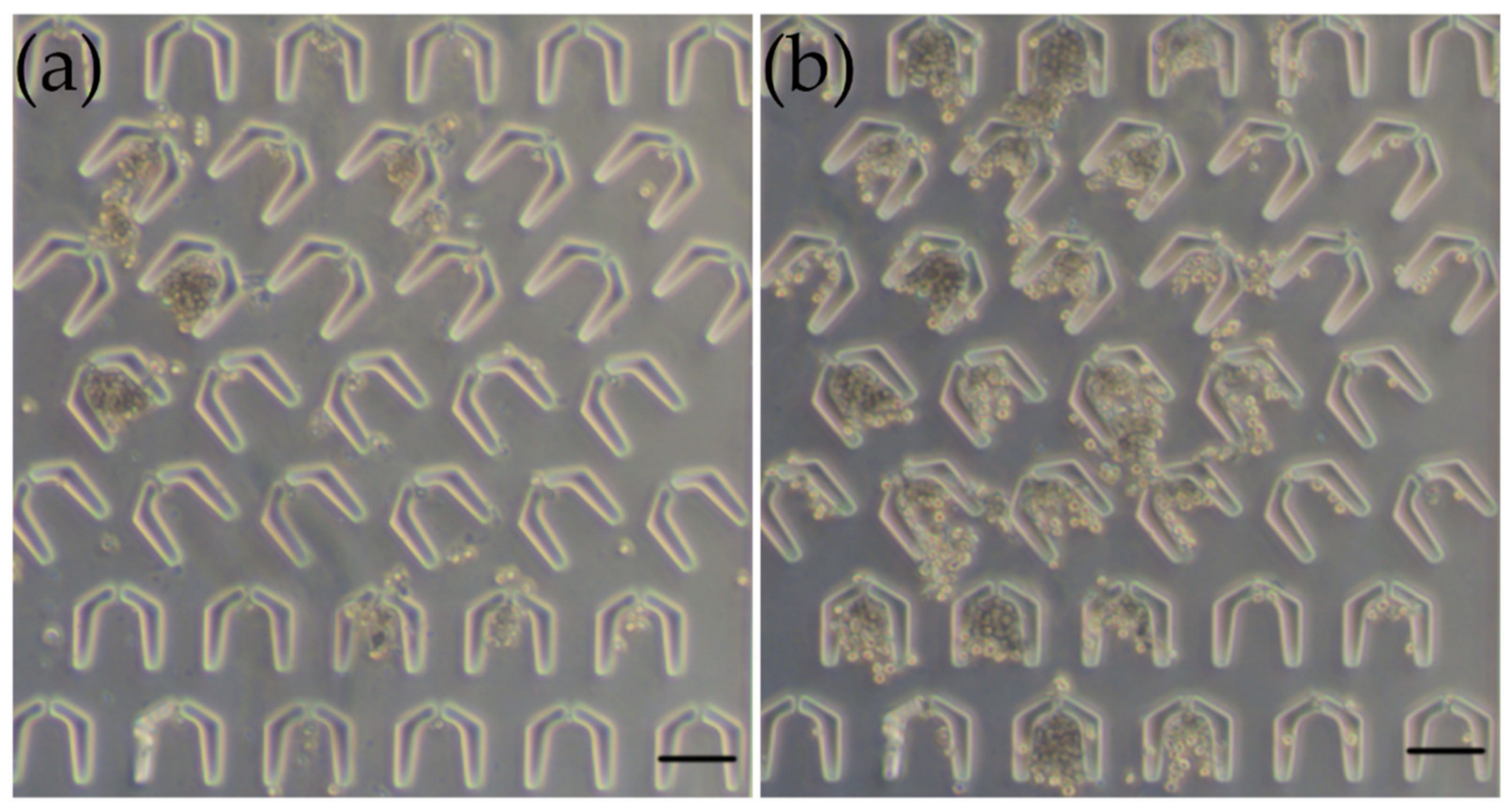
Image of HL-60 cells captured by U-shaped array in microfluidic chip at the end of the reaction. (**a**) Without magnetic field. (**b**) With magnetic field. Scale bar: 100 µm.

Upon preliminary experiments to determine the conditions of cell-probe binding and magnetic capture efficiency, the effect of magnetic capture in the microfluidic chip was then discussed. Observing the capture region of the microfluidic chip, as shown in Figure 8, and comparing it to the applied magnetic field, it was evident that more cells could be captured in the U-shaped array region with the application of the magnetic field. The number of cells at the outlet was measured by calculating the ratio of the number of cells collected at the outlet when the magnetic field was applied to the number of cells obtained when the magnetic field was not applied to obtain the value of the magnetic capture efficiency. According to the calculation, the magnetic capture efficiency of the target cells in the microfluidic chip was 82.92 ± 7.03%, indicating that this microfluidic chip structure combined with magnetic field provided excellent capture of the target cells. The results demonstrate that our method is capable of capturing the target cells with high sensitivity and is comparable to current reports of cell isolation using magnetic particles or other techniques [38,39].

## 4. Conclusions

To sum up, a feasible method of magnetic fluorescent nanoprobes was constructed by stepwise coupling. The addition of PEG not only stabilized the dispersion of the produced probes and minimized non-specific binding, but also added carboxyl sites, which served as a bridge between the antibody and MNP. After the conjugation, the nanoprobes showed strong fluorescence and high selectivity and specificity in labeling CXCR4 sites expressed on the surfaces of HL-60 cells, and the optimal conditions for usage of magnetic fluorescent nanoprobes were also explored. By applying this probe to the microfluidic chip of our design, it provided superior magnetic capture of target cells. Compared with traditional cell separation methods, experiments using microfluidic chips could achieve higher separation efficiency in a shorter separation time with less sample volume. In future work, the magnetic capture of target cells will be more accurate and easier to identify in the future by performing fluorescence quantification on the capture region of the microfluidic chip based on the detection results.

## Figures and Tables

**Table 1:** The coupling quantity and coupling ratio of the antibody and fluorescent molecules.

Sample	Quantities of Coupled Antibody (µg)	Ratio of Coupled Antibody	Quantities of Coupled Fluorescent Molecular (µg)	Ratio of Coupled Fluorescent Molecular
MNP@PEG-12G5-L-F647	46	92%	3.24	27.0%
MNP@PEG-12G5-H-F647	92	92%	7.33	30.5%

## Data Availability

Not applicable.
